# Global Epidemiology of Dementia: Alzheimer's and Vascular Types

**DOI:** 10.1155/2014/908915

**Published:** 2014-06-25

**Authors:** Liara Rizzi, Idiane Rosset, Matheus Roriz-Cruz

**Affiliations:** ^1^Division of Geriatric Neurology, Service of Neurology, “Hospital de Clínicas de Porto Alegre” (HCPA), Ramiro Barcelos Street 2.350, 90035-903 Porto Alegre, RS, Brazil; ^2^Department of Internal Medicine, “Universidade Federal do Rio Grande do Sul” (UFRGS), Ramiro Barcelos Street 2.350, 90035-903 Porto Alegre, RS, Brazil; ^3^Division of Gerontological Nursing, Faculty of Nursing, Universidade Federal do Rio Grande do Sul (UFRGS), São Manoel Street 963, 90620-110 Porto Alegre, RS, Brazil

## Abstract

The prevalence of dementia varies substantially worldwide. This is partially attributed to the lack of methodological uniformity among studies, including diagnostic criteria and different mean population ages. However, even after considering these potential sources of bias, differences in age-adjusted dementia prevalence still exist among regions of the world. In Latin America, the prevalence of dementia is higher than expected for its level of population aging. This phenomenon occurs due to the combination of low average educational attainment and high vascular risk profile. Among developed countries, Japan seems to have the lowest prevalence of dementia. Studies that evaluated the immigration effect of the Japanese and blacks to USA evidenced that acculturation increases the relative proportion of AD cases compared to VaD. In the Middle East and Africa, the number of dementia cases will be expressive by 2040. In general, low educational background and other socioeconomic factors have been associated with high risk of obesity, sedentarism, diabetes, hypertension, dyslipidemia, and metabolic syndrome, all of which also raise the risk of VaD and AD. Regulating these factors is critical to generate the commitment to make dementia a public health priority.

## 1. Introduction

Epidemiological surveys on dementia have two basic points to analyze: the descriptive point, where ratios are calculated for communities and populations included in the study; the analytic point, which attempts to explain phenotypic variations observed by the identification of risk factors [1]. Dementia rates are growing at alarming proportion in all regions of the world and are related to population aging [2]. Neurologic conditions, including dementia, were estimated by the Global Burden of Disease 2010 Study as the third leading cause of years lived with disability at global level [3].

The prevalence of dementia rapidly increases from about 2-3% among those aged 70–75 years to 20–25% among those aged 85 years or more [4]. Over this age, there is paucity of data to affirm whether dementia prevalence keeps increasing or stabilizes [5]. Particularly in very old age, women have slightly greater probability to develop dementia than men, mainly due to an age-adjusted increased risk of Alzheimer's disease (AD) (RR = 1.3) [6]. Whether this gender difference in the prevalence of AD is or is not related to the low number of years of study and intellectual activities among women is still a matter of debate [7]. Conversely, vascular dementia (VaD), as well as stroke and other atherosclerotic cardiovascular diseases, is more prevalent in men [6].

**Figure 1:**
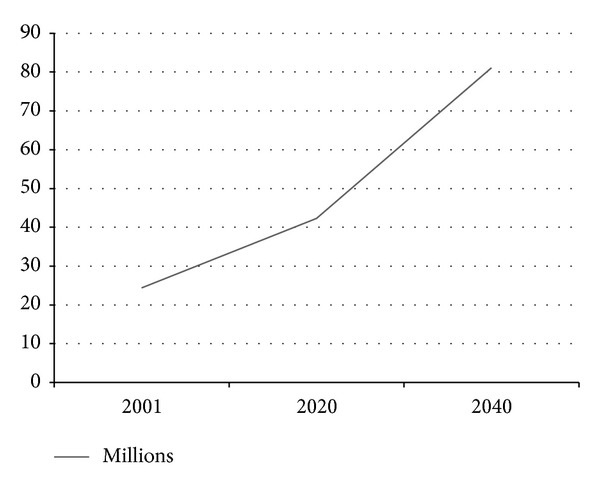
Estimate of numbers of people living with dementia worldwide. Based on raw data from Ferri et al., 2005 [4, page 2114].

Several studies showed that the overall prevalence of dementia varies widely among countries, being influenced by cultural and socioeconomic factors [8]. By 2025, WHO projections suggested that about three-quarters of the population aged 60 or over will be living in developing countries [6]. Another projection indicated that the number of people affected by dementia will double between 2020 (42 million) and 2040 (81 million) (Figure 1) [4].

Alzheimer's disease is the most common type of dementia among western countries, corresponding to about 60% of cases [2, 5], while vascular dementia is the second, with about 20% of all cases. Due to the overlaps in symptomatology, pathophysiology, and risk factors, AD and VaD are not easily distinguished. In fact, VaD refers to the heterogeneous group of clinical syndromes, which include dementia, resulted from ischemic, hemorrhagic, anoxic, or hypoxic brain damage. Ischemic VaD may be due macrovascular or microvascular cerebral disease or yet a combination of both types of lesions. The cause of hemorrhagic VaD may be hypertension, leading to cerebral amyloid angiopathy, source of intralobar hemorrhages, or multiple petechial hemorrhages. In AD, causes and progression are not well understood. The disease is associated with tangles and plaques in the brain, loss of connections, inflammation, and eventual death of brain cells, so it is classified as a neurodegenerative disorder. All these brain changes lead to memory loss and alterations in thinking and other brain functions. The disease usually progresses slowly and gradually gets worse as more brain cells die.

Some studies suggest that the combination of AD and cerebrovascular pathologies corresponds to a type of dementia called mixed dementia [9]. Thereby, each pathology contributes to different levels giving rise to a continuum of patients, in whom pure cerebrovascular disease and pure Alzheimer's disease represent the two extremes of the spectrum [10, 11]. Besides the fact that AD and VaD share a possible common vascular etiopathogeny, the Nun study showed that there is an important neuropathological association between the amyloid/tau proteins and vascular burden in necropsied brain of most individuals with dementia [12]. This and other neuropathological and epidemiological studies led some authors to suggest that the association between AD and VaD pathologies is so common that mixed type dementia may be the most common dementia subtype. The question of how much amyloid/tau proteins and vascular pathology are considered “normal aging” is a matter of debate and has important implications in classifying “pure AD” or “pure VaD” even in neuropathological studies [12].

## 2. Worldwide Prevalence of Dementia 

The distribution of dementia around world seems to vary according to cultural and socioeconomic differences among nations. Interestingly, overall prevalence of dementia in general and AD in particular appears to be higher in developed countries than in developing ones (Table 1). In a review about the global burden of dementia, the higher prevalence in developed countries than in developing was attributed to differences in the level of exposure to cerebrovascular risk factors like hypertension, smoking, obesity, and diabetes [4, 13].

In 2001, 60.1% of all people with dementia were living in developing countries; this proportion is expected to rise to 71.2% by 2040. Aging demographic transition is proceeding rapidly especially in China, India, and Latin America, where dementia is rapidly becoming the major public health problem. Although epidemiological information about the prevalence of dementia and its subtypes remains scarce [2, 4], the Delphi Consensus Study found that the prevalence of dementia was high in Americas and low in less developed regions of the world, such as Africa and the Middle East. Prevalence of dementia in Latin America will be similar to that encountered in North America by 2040 [4]. Among developed countries, Japan has the lowest prevalence of both dementia in general and Alzheimer's disease in particular. And dementia prevalence in Eastern European countries was relatively uniform.

The paragraphs below and Table 2 summarize epidemiological studies on dementia conducted in various regions of the world by comparing the relative prevalence of AD and VaD.

### 2.1. European Studies

A collaborative study of European population-based cohorts identified the total of 2346 cases of mild to severe dementia in 11 cohorts. Age-standardized prevalence was 6.4% for dementia (all causes), 4.4% for AD, and 1.6% for VaD [14].

The Rotterdam Study estimated the prevalence of dementia and its subtypes in Ommoord, suburb of Rotterdam, and examined the relationship of the disease expressions with educational level. The results showed high prevalence of overall dementia and AD in subjects with low levels of education. Although dementia was associated with high levels of atherosclerosis, the effect of low educational status was not modified by adjusted cardiovascular risk factors [15, 25].

A study on the incidence rates in UK showed that the incidence for AD was 1.59/1000 person-years (95% CI 1.55–1.62) and for VaD was 0.99/1000 (95% CI 0.96–1.02). The incidence for AD was higher for women than for men but not for VaD. They yet found that demented patients with cardiovascular diseases may be likely diagnosed with VaD more than AD [26].

In Italy, the ILSA Study estimated that the average incidence rates per 1000 person-years were 12.47 for overall dementia, 6.55 for AD, and 3.30 for VaD. They yet found out that women carry high risk of developing AD, whereas men carry high risk of developing VaD [16, 27].

### 2.2. North American Studies

It is estimated that 5.2 million old Americans have Alzheimer's disease, that is, one in nine individuals aged 65 years or older (11%) [28], and the majority are women, probably because women generally live longer than men. AD is already the sixth leading cause of all deaths in USA and the fifth cause among Americans aged more than 65 years [29]. Based on the Aging Demographics and Memory Study (ADAMS) about 14% of people aged more than 71 years in USA have dementia [30]. Dramatic increases in the number of people aged more than 85 years across all racial and ethnic groups will affect the number of people living with AD in the next years [7]. In USA, by 2030 the number of patients with AD will increase 50%, and by 2050 it may nearly triple [7]. About 450 thousands older Americans with AD died in 2013, a large proportion as result of complications of AD [29]. Data indicate that, in USA, older blacks are twice as likely to have AD and other dementias than older whites [31], showing the racial differences in this country.

### 2.3. Latin American Studies

The prevalence of dementia in Latin America is almost similar to that encountered in North America. This phenomenon may be due to the special combination of low average educational attainment and high vascular risk profile among Latin American elderly populations.

In fact the Delphi Consensus Study found that, after the age of 75, the prevalence of dementia in the poorest regions of Latin America was higher than in other developed regions of the world [4]. Nitrini and coworkers observed that the rate of illiteracy among elderly Latin Americans was 9.3% and that the prevalence of dementia (15.7%) was two times higher than in literates (7.16%). This phenomenon tries to explain that low educational attainment leads to diminished cognitive reserve [32].

So, if socioeconomic deprivation is associated with increased load of cerebrovascular disease, it cannot be underestimated [33]. Latin America, by 2040, will have many people with dementia as North America, 9.1 million and 9.2 million, respectively [4].

### 2.4. Asian Studies

The burden of dementia is increasing exponentially especially in Asia-Pacific region, where more than 60% of the population reside [34]. The prevalence of dementia seems to be higher in developed countries, like Japan and Korea, than in countries with low incomes in Asia. A Japanese study found that the prevalence of dementia equals 11% among those aged more than 65 years [35, 36], whereas a Korean one found the prevalence of 6.3% [17]. Another study conducted in Korea, the Seoul study, showed that the prevalence of dementia, excluding very mild cases, was about 5.3% for overall dementia and 4.3% for AD [37].

The prevalence of dementia greatly varies between different ethnic groups living in the same country, like in Singapore, that is probably the most multicultural region of Asia. A Singaporean study showed low standardized dementia prevalence among the ethnic Chinese (2.5% among the elderly) when compared to the ethnic Malays (4.0% among the elderly) and this finding was independent of the frequency of vascular risk factors [18]. Whereas these differences are due to different genetics or lifestyle it remains a matter of debate.

#### 2.4.1. Chinese Studies

At the most populous country of the world, aging population is occurring in a wide scale. In 2000, 10% of the total China population had 60 years or more and by 2050 one in four Chinese people will be aged more than 65 years. Compared with Western countries and Caucasian populations, studies in China suggest low prevalence of overall dementia (average 3% in different studies) [19, 20]. A meta-analysis showed that the prevalence in a population aged 60 years or older for AD was 1.9% and for VaD was 0.9% [21]. Higher prevalence numbers were concentrated in metropolitan cities and in provinces near east coast. Nowadays, the estimated number of older people with dementia in mainland China, Hong Kong, and Taiwan together is about 8.4 millions [38].

Just like in Japan few decades ago, VaD is more common in China than AD [19]. However, some studies conducted in Southern China have already found that AD is becoming more prevalent than VaD. In 2004, the prevalence of AD in adult population was 2.0% and 1.2% in Southern and Northern China, respectively. Conversely, prevalence of VaD was 0.6% and 1.1%, respectively [20]. In China, AD becomes more common than VaD since 1990. Other surveys, in contrast, exhibit that the current prevalence of dementia subtypes could be compared with western and developed countries [21, 39, 40].

#### 2.4.2. Japanese Studies

Among developed countries, Japan seems to have the lowest prevalence of dementia in general and Alzheimer's disease in particular. Traditionally, VaD used to be more predominant in Japan than AD [41, 42]. The Hisayama Study determined the type-specific incidence of dementia and risk factors in Japanese population. The age-adjusted total incidence (per 1000 person-years) of dementia in the period of 1985–1992 was 19.3 for men and 20.9 for women. Corresponding rates for vascular dementia were 12.2 for men and 9.0 for women, whereas for AD they were 5.1 for men and 10.9 for women. Multivariate analysis showed that age, prior stroke episodes, systolic blood pressure, and alcohol consumption were independent risk factors for VaD; meanwhile, age was significant risk factor of AD [43].

A recent survey with the same community, followed up prospectively for 17 years, showed that incidences (1000 person-years) were overall dementia 32.3, AD 14.6, VaD 9.5, dementia of Lewy bodies 1.4, mixed type 3.8, and other types 3.1. The incidence of AD, mixed-type dementia, and other types of dementia increased with age, particularly after 85 years, but this tendency was not observed for dementia of Lewy bodies [44].

In general, secular trends in the age-adjusted prevalence of all causes of dementia and AD significantly increased in Japanese population over the past two decades, and the ratio of VaD to AD decreased with the time [45, 46]. Analyzing associations with blood pressure and dementia, the age- and sex-adjusted incidence of VaD significantly increased with elevated midlife and late-life hypertension levels, but it did not occur for AD. Midlife hypertension was strongly associated with greater risk of VaD, regardless of late-life blood pressure levels [46, 47]. In fact, midlife systolic blood pressure is the strongest blood component to predict incident dementia [48]. Therefore, the better hypertension control was possibly the main reason for decreased prevalence of VaD in Japan. On the other hand, population aging and westernization of lifestyle were probably the main determinants for increased prevalence of AD in Japan.

In the last three decades Japan VaD/AD prevalence ratio decreased from 2 : 1 to 1 : 1. Changes in lifestyle such as reduced salt intake and treatment of hypertension seem to be the main causes of the reduced incidence of both stroke and VaD. However, increased life expectancy and westernization of lifestyle, including diet, might also have contributed to the increased prevalence of AD in this country, consequently reducing the VaD/AD ratio. This hypothesis is in accordance with the vascular hypothesis for AD, by which other vascular risk factors than hypertension may increase the risk of AD compared to that of VaD itself. Consistent with this, old Japanese immigrants in Hawaii and California tend to have an in-between risk profile for both AD and VaD, when compared with the Japanese living in Japan and Americans living in USA [42].

*Cross-Cultural Japanese Studies*. Findings from Honolulu-Asian Aging Study suggested that the Japanese living in USA have higher prevalence of AD than those living in Japan [42]. Prevalence in Hawaii was similar to Caucasian American populations [49] and the distribution of dementia subtypes closely resembled that found in Caucasian populations in both North America and Europe [50].

An interesting survey associated high prevalence rate of dementia in Japanese-Brazilians from Okinawa when compared with the Japanese living in Okinawa with dietary factors. The dietary pattern of Japanese immigrants in Brazil, characterized by low fish consumption and large meat intake, increased the prevalence of dementia among immigrants [51].

The disparities in prevalence rates of dementia subtypes are especially apparent in studies comparing eastern and western countries. However, such large variation might be at least partially explained by methodological differences between studies [52]. For instance, prevalence of dementia among different Japanese population samples aged 65 or over ranges from 3.8% to 11%. Furthermore, the AD/VaD ratio in these populations ranged from 2.1% to 4.1% [36]. Therefore, considering the difficulties involved in establishing diagnostic threshold for dementia as well as incomplete population mean age adjustments, the actual differences in overall dementia prevalence among Asian populations, even as between them and western countries, are probably lower than believed [37].

### 2.5. African Studies

Longevity in Africa nowadays is increasing quickly in absolute numbers and dementia is normally dismissed as part of normal aging. In addition, the later diagnostic in the disease process, when done, and less factual information about the disease have been associated with significantly higher dementia-related morbidity and health care costs. The difficulty to obtain and organize real rates in Africa could be explained by different survival rates, the hiding of cases, reluctance to seek medical assistance, poor access to medical care, and defective case-finding techniques [53].

A systematic analysis in Africa estimated the overall prevalence of dementia in people older than 50 years in about 2.4% (2.76 million people), the majority of them living in Sub-Saharan Africa (76%). That is the area that has the highest disease burden in the world and is the only region of the planet where it is expected that the number of miserable people will increase in next years. Prevalence in general was the highest among females aged 80 or over (19.7%) and there was little variation between regions. AD was the most prevalent cause of dementia (57.1%) followed by VaD (26.9%) [23].

A study conducted in Sub-Saharan Africa compared the prevalence rates of dementia using the DSM-IV criteria with those obtained using the 10/66 diagnostic criteria, which is specifically designed to use in low- and middle-income countries. The results showed the age-standardized prevalence of clinical DSM-IV dementia was about 6.4% and for 10/66 dementia it was about 21.6%. In that region, the association between educational level and dementia using the 10/66 criteria was the result of an educational bias within the diagnostic instrument. In this case, the DSM-IV criteria represented an international standard for dementia diagnosis [54].

The Indianapolis-Ibadan Dementia Project compared the incidence rates of Alzheimer's disease in two culturally diverse but genetically related Yoruba elderly community-dwelling populations: one is in Ibadan, Nigeria, and the other is African American in Indianapolis, Indiana. The results showed that age-standardized annual incidence rates were significantly lower among native Yoruba than among African Americans for both overall dementia (2.3% and 8.3%, resp.) and AD (1.4% and 6.3%, resp.) [22, 55]. Of note, the increased prevalence of dementia in aforementioned Africa-American community (6.0%) was mainly attributed to higher prevalence for AD (more 4.9%), as compared with a smaller increase in the prevalence of other causes of dementia (1.1%), including VaD. Like in Japan [42], the relative ratio of AD to other causes of dementia was higher in USA (3.2 : 1.0) than in Nigeria (1.6 : 1.0). In fact the better source of evidence for the importance of environmental factors as opposed to ethnic factors in determining prevalence of dementia comes from the ecological studies above, although there were large discrepancies in dementia prevalence rates depending on which diagnostic system was used.

Interestingly, in the Nigeria-USA study, the prevalence of dementia was higher in USA despite the better educational system. These results do not necessarily argue against the education-related “cognitive reserve” hypothesis in dementia [56] but may imply that other environmental factors, such as diet and physical activity, might be even more important risk factors for dementia than low educational background.

### 2.6. Middle East

Epidemiological studies in AD and VaD have been rarely reported in the Middle East and the most were from Israel. A survey on a rural area of Wadi Ara, Israel, showed that more than 20.5% of the elderly population, the most illiterate, was diagnosed with AD. The annual incidence of AD among those with mild cognitive impairment was 4.4%. VaD constituted about 22% of the cases of dementia and it was strongly associated with illiteracy and hypertension. These findings were considered unique due the high frequency rates of dementia [24]. A study highlighted that the prevalence of cognitive impairment and AD was unusually high in Wadi Ara, while the rate of conversion from cognitive impairment to AD was low [57]. The proportionate increase in people number with dementia in this region and North Africa will be about 300% in 2040 [4], more than in developed countries that will be about 100%.

## 3. Final Remarks

The prevalence of dementia, particularly AD, is increasing fast in both developing and developed countries; however, it varies substantially worldwide. The Delphi Consensus Study evidenced that the prevalence of dementia was higher in Americas and lower in less developed regions of the world, such as Africa and the Middle East. In Eastern European countries it seems to be relatively uniform, lying between Japan and USA. This variation is partially attributable to the lack of methodological uniformity among studies, including diagnostic criteria.

Different mean population ages may be source of heterogeneity, even when considering only elderly populations. In general, countries with high life expectancies tend to have higher proportion of oldest-old individuals and lower proportion of youngest-old than countries with low life expectancies. Standardized age adjustment was not conducted in many epidemiological studies on dementia. Anyway, even after considering these potential sources of bias, small but substantial difference in age-adjusted prevalence of dementia still exists in different regions of the world.

The universal use of a standardized international epidemiological method is also a* sine qua non* condition to compare the true prevalence of both AD and VaD in the different regions of the world. Besides unequal methodologies and life expectancies, differences in diet and physical activity at one side and education attainment at another side explain most of the disparities in prevalence of dementia around the different regions. And even though education attainment protects against dementia [56], increased cardiovascular risk factors like hypertension, diabetes, obesity, and dyslipidemia seem to counterweight the dementia towards a higher prevalence in developed countries.

On the other hand, developing societies where hypertension is the major problem seem to have proportionally high prevalence of VaD. Development, being in western or eastern countries, increased the prevention, treatment, and control of hypertension (theory of morbidity compression) [58], even if the prevalence of obesity keeps rising. With the socioeconomic development and high life expectancy, the prevalence of AD rises, thus increasing the AD/VaD rate.

Whichever for AD or VaD, to date the best way to implement public policies in order to decrease the burden of dementia upon societies is by improving education of children and adults and fostering healthy environments and lifestyles, including balanced diet and the regular practice of exercise.

## Figures and Tables

**Table 1:** Worldwide estimate for the absolute number of cases of dementia according to the Delphi Consensus Study.

	Absolute number of people over 60 years old who have dementia (millions)		
	2001	2020	2040
Western Europe	4.9	6.9	9.9
Eastern Europe low adult mortality	1.0	1.6	2.8
Eastern Europe high adult mortality	1.8	2.3	3.2
North America	3.4	5.1	9.2
Latin America	1.8	4.1	9.1
North Africa and Middle Eastern Crescent	1.0	1.9	4.7
Developed Western Pacific	1.5	2.9	4.3
China and the developing Western Pacific	6.0	11.7	26.1
Indonesia, Thailand, and Sri Lanka	0.6	1.3	2.7
India and South Asia	1.8	3.6	7.5
Africa	0.5	0.9	1.6

Total	24.3	42.3	81.1

Created from raw data provided by Ferri et al., 2005 [4, Page 2115].

**Table 2:** Summary of some studies that evaluate the prevalence of dementia in respective countries or regions.

Country/ region	Study	Year	AD	VaD	Prevalence of dementia	Reference
Europe	Collaborative study	1990	4.4% > 65 years	1.6% > 65 years	6.4% > 65 years	[14]

Ommoord	Rotterdam Study	1995	72% > 55 years	16% > 55 years	6.3% > 55 years	[15]

Italy	ILSA Study	1992-1993	6.55% > 65 years	3.30% > 65 years	12.47% > 65 years	[16]

USA	ADAMS	2007	—	—	14% > 71 years	[7]

Latin America	Delphi Consensus Study	2005	—	—	7.3% > 75 years	[4]

Korea	KLoSHA	2006	4.8% > 65 years	1.0% > 65 years	6.3% > 65 years	[17]

Singapore	Singaporean study	1995	—	—	Chinese: 2.5% and Malays: 4.0% among the elderly	[18]

China	People's Republic of China study	1980–2010	1.9% > 60 years	0.9% > 60 years	3.0% > 60 years	[19–21]

Africa-USA	Indianapolis-Ibadan Dementia Project	2001	Native Yoruba: 1.4%, African American: 6.3%	—	Native Yoruba: 2.3%, African American: 8.3%	[22]

Africa	Systematic analysis	2012	57.1% > 50 years	26.9% > 50 years	2.4% > 50 years	[23]

Middle East	Wadi Ara study	2002	20.5% among the elderly	22.0% among the elderly	—	[24]
